# Genomic Recombination Leading to Decreased Virulence of Group B *Streptococcus* in a Mouse Model of Adult Invasive Disease

**DOI:** 10.3390/pathogens5030054

**Published:** 2016-08-05

**Authors:** Sarah Teatero, Paul Lemire, Ken Dewar, Jessica Wasserscheid, Cynthia Calzas, Gustavo V. Mallo, Aimin Li, Taryn B.T. Athey, Mariela Segura, Nahuel Fittipaldi

**Affiliations:** 1 Public Health Ontario Laboratory, 661 University Avenue, Suite 17-100, Toronto, ON M5G 1M1, Canada; sarah.teatero@oahpp.ca (S.T.); gustavo.mallo@oahpp.ca (G.V.M.); aimin.li@oahpp.ca (A.L.); taryn.athey@oahpp.ca (T.B.T.A.); nahuel.fittipaldi@oahpp.ca (N.F.); 2 Laboratory of Immunology, Faculty of Veterinary Medicine, University of Montreal, 3200 Sicotte Street, Saint-Hyacinthe, QC J2S 2M2, Canada; paul.lemire@umontreal.ca (P.L.); cynthia.calzas@umontreal.ca (C.C.); mariela.segura@umontreal.ca (M.S.); 3 McGill University and Genome Quebec Innovation Centre, 740 Dr. Penfield Avenue Rm 7104, Montreal, QC H3A 0G1, Canada; ken.dewar@mcgill.ca (K.D.); jessica.wasserscheid@mail.mcgill.ca (J.W.); 4 Department of Laboratory Medicine and Pathobiology, Faculty of Medicine, University of Toronto, 27 King’s College Circle, Toronto, ON M5S 1A1, Canada

**Keywords:** group B *Streptococcus*, *Streptococcus agalactiae*, recombination, invasive bacterial infection, adult infection, cytokines

## Abstract

Adult invasive disease caused by Group B *Streptococcus* (GBS) is increasing worldwide. Whole-genome sequencing (WGS) now permits rapid identification of recombination events, a phenomenon that occurs frequently in GBS. Using WGS, we described that strain NGBS375, a capsular serotype V GBS isolate of sequence type (ST)297, has an ST1 genomic background but has acquired approximately 300 kbp of genetic material likely from an ST17 strain. Here, we examined the virulence of this strain in an in vivo model of GBS adult invasive infection. The mosaic ST297 strain showed intermediate virulence, causing significantly less systemic infection and reduced mortality than a more virulent, serotype V ST1 isolate. Bacteremia induced by the ST297 strain was similar to that induced by a serotype III ST17 strain, which was the least virulent under the conditions tested. Yet, under normalized bacteremia levels, the in vivo intrinsic capacity to induce the production of pro-inflammatory cytokines was similar between the ST297 strain and the virulent ST1 strain. Thus, the diminished virulence of the mosaic strain may be due to reduced capacity to disseminate or multiply in blood during a systemic infection which could be mediated by regulatory factors contained in the recombined region.

## 1. Introduction

Group B *Streptococcus* (GBS, also known as *Streptococcus agalactiae*) is a major agent of neonatal infections and also causes invasive disease in adults [1]. GBS strains are classified into 10 serotypes (Ia, Ib, and II-IX), based on an antigenic reaction directed against the capsular polysaccharide [2,3]. Historically, an overabundance of serotype III strains has been observed among neonatal infections [4,5]. On the other hand, the increase in adult GBS infections observed in the last decades has been driven mainly by serotype V GBS strains [4,5,6]. A multi-locus sequence typing (MLST) scheme is widely used to classify GBS strains [7]. More than 700 sequence types (STs) have been described, grouped into a relatively small number of major genetic lineages or clonal complexes (CCs) [8]. However, only a few successful GBS clonal complexes are associated with human infections world-wide [9].

Recombination involving vast areas of the GBS genome occurs frequently [10,11,12]. For example, strains of ST17 (the founder ST of CC17) associated with neonatal invasive disease were, until recently, exclusively typed as serotype III [13]. However, we and others have described exchange of DNA material including genes involved in capsule biosynthesis in strains of serotype III CC17, giving rise to serotype IV CC17 strains [14,15]. The surface adhesin HvgA, associated with GBS neonatal meningitis [16], was until very recently considered unique to CC17 strains. However, we recently discovered that a non-CC17 strain (NGBS375) has acquired a genomic region of approximately 300 Kbp containing gene *hvgA* and other genes encoding key GBS virulence factors [15]. Strain NGBS375 is a serotype V isolate belonging to ST297 (a member of CC1). It has been shown that the vast majority of invasive serotype V GBS strains causing bacteremia in non-pregnant adults belong to ST1, the founder ST of CC1 [17].

Here, we tested the hypothesis that recombination confers this ST297 strain with a unique virulence potential which impacts disease pathogenesis. We report that genomic recombination results in impaired ability of the ST297 isolate to induce systemic disease in a mouse model of adult invasive disease.

## 2. Results

### 2.1. Genome Comparisons Show that Strain NGBS375 Has an ST1 Genome Backbone but Has Acquired an Area of 308,916 bp by Homologous Recombination from an ST17 Donor

**Figure 1:**
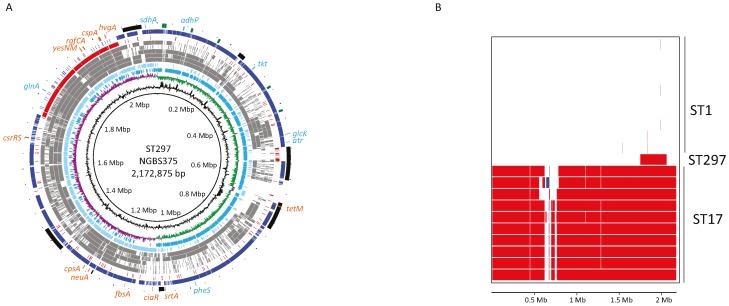
The genome of strain NGBS375 is a mosaic of ST1 and ST17 genomic content. (**A**) Genome atlas of strain NGBS375 (ST297). Depicted data from innermost to outermost circles represent genome size in Mbp (circle 1), percent G+C content (circle 2), GC skew, or (G-C)/(G+C), averaged over a moving window of 10,000 bp, with excess G and excess C shown in green and purple, respectively (circle 3). Circle 4 shows annotated coding sequences (CDSs) on the forward/positive-strand (**dark blue**), while circle 5 shows reverse/negative-strand encoded CDSs (**light blue**). Distribution of SNPs identified in strains NGBS572, NEM316, A909, NGBS061, and 2603VR, relative to the genome of strain NGBS375 (ST297) are shown in grey in circles 6, 7, 8, 9, and 10, respectively. Circle 11 shows SNPs identified in ST1 strain NGBS357 (**red**) relative to the ST297 strain. Circle 12 shows SNPs identified in ST17 strain NGBS128 (**blue**) relative to the ST297 strain. Reference landmarks are shown in circle 13: Mobile genetic elements are depicted in **black**; genes used in the GBS MLST scheme are shown in **light blue**; *hvgA* gene and other genes of interest, in **orange**; (**B**) Areas of recombination based on the genomes of ten ST1 GBS strains, ten ST17 GBS strains, and mosaic NGBS375 (ST297). Each horizontal band represents a bacterial strain. The panel shows a horizontal representation of the recombinant segments that were predicted for each strain. The horizontal scale represents the length of the NGBS375 genome. Colors are arbitrarily assigned; fragments of the same color and in the same column are from the same origin across different strains. The area of recombination in NGBS375 (shown in **red**) is from genome position 1,750,311 to 2,059,227 bp. ST17 strains used: NGBS317, NGBS398, NGBS169, NGBS470, NGBS299, NGBS500, NGBS534, NGBS291, NGBS238, and NGBS636. ST1 strains used: NGBS180, NGBS246, NGBS444, NGBS267, NGBS283, NGBS303, NGBS348, NGBS380, NGBS425, and NGBS558.

Our previous data suggested that the genome of strain NGBS375 (ST297) may have acquired a large genomic region from a putative CC17 donor [15]. To study the issue in more detail, we sequenced to closure the genome of strain NGBS375, as well as the genome of one arbitrarily chosen strain of ST1 (NGBS357). We next compared the genome of NGBS375 (ST297) to that of NGBS357 (ST1) and to the genomes of several other GBS strains representing seven other STs. For most STs, we identified >10,000 single-nucleotide polymorphisms (SNPs) relative to the genome of strain NGBS375 (ST297) (Table 1). However, we noticed that for strain NGBS128 (ST17), these polymorphisms were unevenly distributed, with a low density of SNPs (fewer than 100 unique polymorphisms) mapping between positions 1,760,000 and 2,060,000 bp of the NGBS375 (ST297) genome (Figure 1A). On the other hand, in strain NGBS357 (ST1), this area had a high concentration of SNPs, but few SNPs mapped to other regions of the NGBS375 (ST297) genome (Figure 1A and Table 1). These data strongly suggested that NGBS375 possesses an ST1 genomic background but has acquired a large block of ST17 gene content. Using Bayesian analysis of recombination [18], we precisely defined a 308,916 bp region having undergone recombination in the genome of the mosaic ST297 strain (nucleotides 1,750,311–2,059,227) (Figure 1B).

### 2.2. Genetic Content in the Recombined Region of ST297Strain NGBS375

The area of recombination in ST297 strain NGBS375 contains 301 genes, including several encoding known and putative virulence factors, membrane-associated proteins and transcriptional regulators (Supplementary Table S2). In addition to *hvgA*, the gene encoding a adhesin implicated in invasion of epithelial barriers [16], this region contains a gene encoding a C3 degrading proteinase, the gene encoding the immunomodulatory protein GAPDH [19,20], and gene *cspA*, encoding a serine protease that inactivates CXC chemokines [21]. Of particular interest, this region contains 19 genes predicted to encode transcriptional regulators, including *rgfCA*, a two-component system that mediates GBS binding to extracellular matrix components such as fibrinogen [22].

### 2.3. Ortholog Analysis of GBS Strains

**Figure 2:**
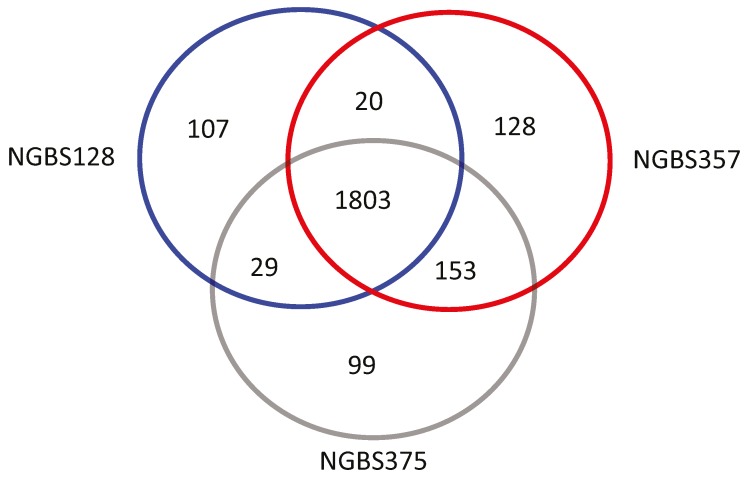
Orthologous gene clusters identified in the genome of strains NGBS357 (ST1), NGBS128 (ST17), and NGBS375 (ST297). PGAP was used to identify orthologous gene clusters between strains. Numbers of shared gene clusters are shown in overlapping areas.

Although NGBS375 is a mosaic of ST1 and ST17 strains, it is highly unlikely that it is derived directly from the ST1 and ST17 strains used here for genome comparison. That is, other differences in gene content may exist between the three strains. We assessed this hypothesis by performing ortholog gene cluster analysis. Genome annotation predicted 2090 protein-coding sequences (CDSs), 80 tRNA genes, and 21 rRNA genes in strain NGBS375 (ST297), while 2155 CDSs, 80 tRNA genes, and 21 rRNA genes were predicted for strain NGBS357 (ST1). ST17 strain NGBS128 had 21 rRNA and 80 tRNA-encoding regions, and 1981 CDSs [23]. PGAP analysis revealed 1803 orthologous gene clusters common to all three strains (Figure 2). There were 99 gene clusters unique to NGBS375 (ST297), 107 gene clusters unique to NGBS128 (ST17) and 128 gene clusters unique to strain NGBS357 (ST1) (Supplementary Tables S3–S5). The majority of unique gene clusters in each strain were associated with mobile genetic elements (MGE) (see Supplementary Tables S3–S5). Interestingly, the ST1 strain also possessed the erythromycin resistance determinant *ermTR*, and the MGE-associated virulence factor *esxA* [24].

### 2.4. Altered Virulence of the Mosaic GBS Strain in An Adult Mouse Model of Systemic Infection

**Figure 3:**
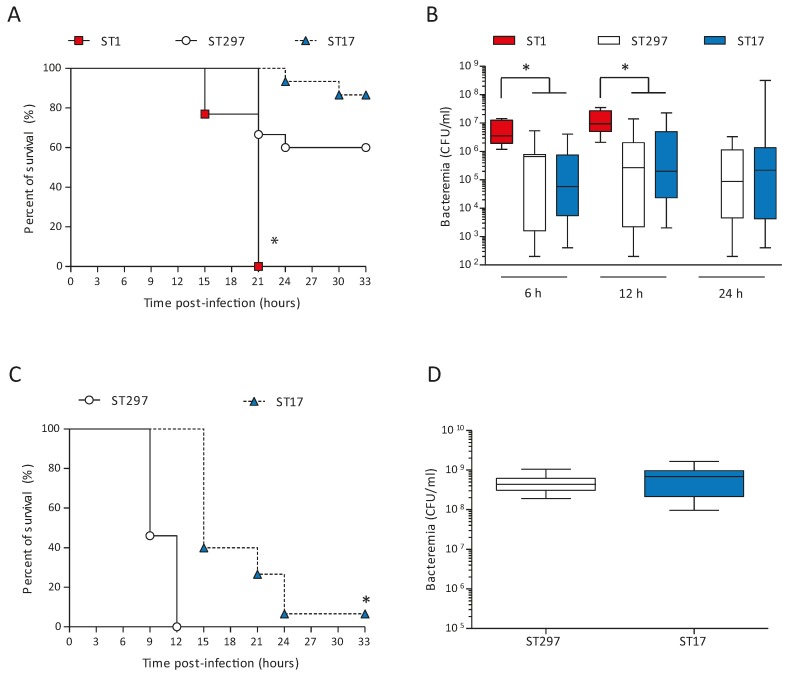
Susceptibility of mice to intraperitoneal infection with GBS strains NGBS128 (ST17), NGBS357 (ST1), and NGBS375 (ST297). (**A**) Survival curves of C57BL/6 mice (*n* = 15 per group) infected using the intraperitoneal route with 1 × 10^5^ CFU of GBS strains. There was a significant difference (* *p* < 0.0001) in survival between the ST297 strain NGBS375 and the ST17 strain NGBS128 versus the ST1 strain NGBS357 according to the log-rank test (Mantel-Cox). No significant differences were observed between the ST297 and the ST17 strains; (**B**) Blood bacteremia of mice infected as described in (**A**). For 6 h and 12 h, there was a significant difference (* *p* < 0.001) between the ST297 strain and the ST17 strain versus the ST1 strain according to the ANOVA test. No significant differences were observed between the ST297 strain and the ST17 strain. No data were available for the ST1 group at 24 h because all mice had succumbed to the infection before 24 h; (**C**) Survival curves of C57BL/6 mice infected with 1 × 10^7^ CFU of GBS strains (*n* = 15). There was a significant difference (* *p* < 0.0001) between the ST297 strain and the ST17 strain according to the log-rank test (Mantel-Cox); (**D**) Blood bacteremia of mice infected as described in (**C**). No significant differences were observed between the ST297 and the ST17 strains. For (**B**) and (**D**) blood samples were collected from the tail vein at the indicated time p.i., and plated onto THB agar plates. Colonies were enumerated and data expressed as CFU/mL of blood. Data are displayed as box and whisker plots. The horizontal line represents the median, the box represents the interquartile range and the whiskers represent the range.

Acquisition or exchange of large areas of genome content can greatly impact several traits of bacterial pathogenic organisms, including virulence. To begin to investigate how genetic exchange affects the virulence of the ST297 isolate, we performed experimental infections of adult mice. As comparators, we used one ST1 strain (NGBS357) and one ST17 strain (NGBS128). All mice infected with the ST1 strain died by 21 h post-infection (p.i.) (Figure 3A). On the other hand, 60% of mice survived the infection in the ST297 group (*p* < 0.0001 vs. ST1-infected mice). At a dose of 10^5^ CFU, the survival curve of ST297-infected mice was similar to the one observed in mice infected with the ST17 strain (*p* > 0.05). In agreement with these results, bacteremia levels at 6 and 12 h p.i. were similar in these two groups, but significantly lower than those observed in animals infected with the highly virulent ST1 strain (Figure 3B). To better dissect possible differences between the ST297 and the ST17 strains, mouse mortality was monitored after infection with a higher bacterial dose (10^7^ CFU). Under these conditions, mice infected with the ST297 strain showed significantly greater mortality levels than animals infected with the ST17 strain (*p* < 0.0001; Figure 3C), with 100% mortality at 12 h p.i. Yet, at 6 h p.i., bacteremia levels were similar between the two groups (Figure 3D). Taken together, these data indicate that the mosaic ST297 strain has an intermediate virulence between ST1 and ST17 strains, but induces bacteremia levels comparable to the ST17 strain.

### 2.5. In vivo Systemic Inflammatory Response induced by the ST297 GBS Strain

**Figure 4:**
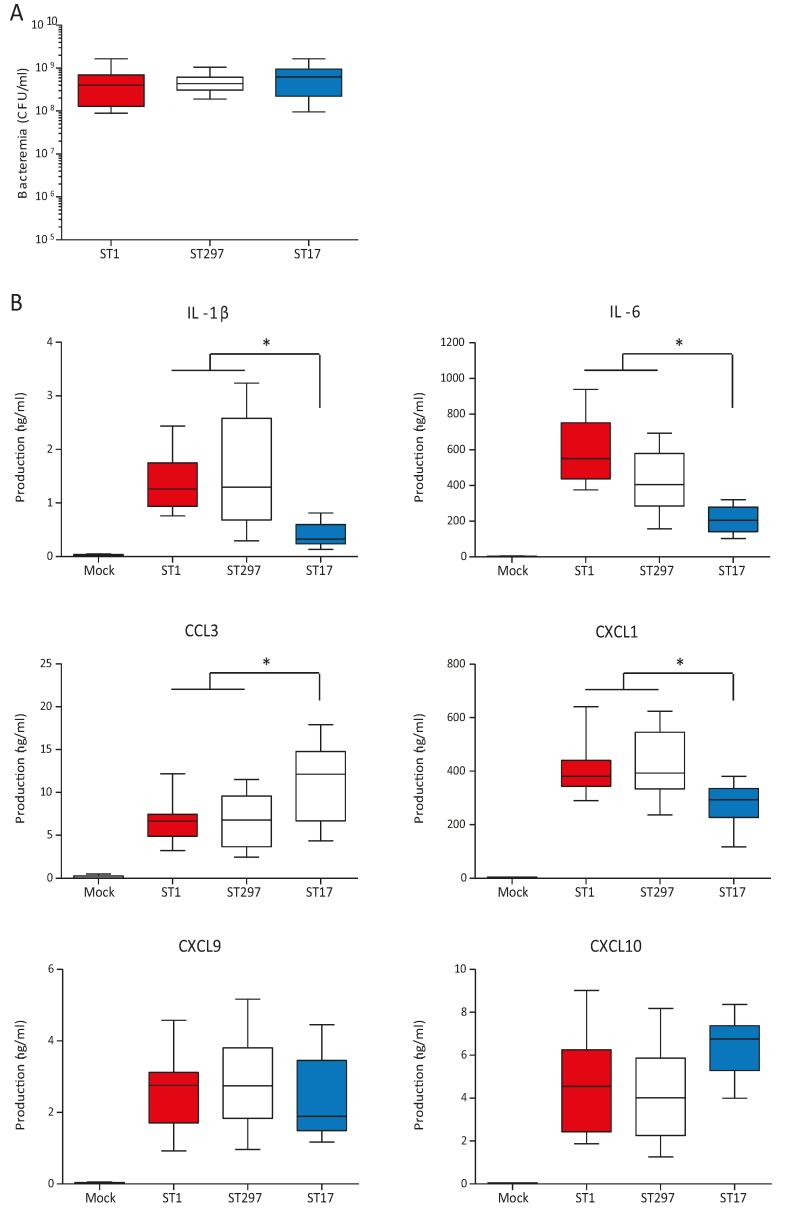
Plasma levels of pro-inflammatory cytokines in mice infected with GBS strains NGBS128 (ST17), NGBS357 (ST1), and NGBS375 (ST297). C57BL/6 mice were infected using the intraperitoneal route with 1 × 10^7^ CFU of GBS strains, and euthanized at 6 h p.i.. Mock-infected mice (vehicle solution only) were used as non-infected controls. (**A**) Bacteremia in mice infected as previously described. Blood samples were collected by cardiac puncture, and plated onto THB agar plates. Colonies were enumerated and data expressed as CFU/mL of blood. Data are displayed as box and whisker plots. The horizontal line represents the median, the box represents the interquartile range and the whiskers represent the range. No significant differences were observed between the strains; (**B**) Plasma was collected and production of IL-1β, IL-6, CCL3, CXCL1, CXCL9 and CXCL10 was measured by ELISA. Data are displayed as box and whisker plots from two independent experiments (total *n* = 20; 10 mice per group, per experiment). The horizontal line represents the median, the box represents the interquartile range and the whiskers represent the range. * *p* < 0.05 indicates statistically significant differences between the ST1 strain, and the ST297 strain, versus the ST17 strain according to ANOVA test. No significant differences were observed between the ST1 strain and the ST297 strain.

Levels of plasma cytokine production are directly influenced by levels of bacteremia [25]. Thus, to be able to accurately compare the intrinsic capacity of different GBS strains to induce inflammatory mediators, we used a similar infective dose for all three strains (Figure 4A). Under these conditions, mice infected with the ST297 strain showed similar plasma levels of key inflammatory cytokines and chemokines (such as IL-1β, IL-6, CCL3, CXCL1, CXCL9, and CXCL10) as those observed in mice infected with the ST1 strain (*p* > 0.05) (Figure 4B). In contrast, mice infected with the ST17 strain showed reduced levels of IL-1β, IL-6 and CXCL1 compared to ST297- and ST1-infected mouse groups (*p* < 0.05). Interestingly, infection with the ST17 strain resulted in higher plasma levels of the chemokine CCL3 than those observed in the other groups.

## 3. Discussion

Here, we confirm that strain NGBS375, a previously described serotype V ST297 strain isolated from a case of adult invasive GBS infection, has a *bona fide* CC1 genome background, but has acquired a large genetic region from a presumed CC17 donor by means of recombination. The genome sequence of this strain is now available (NCBI accession number CP102503). Among other important exchanged virulence factors, the mosaic strain acquired the gene encoding the virulence factor HvgA. This surface-exposed protein mediates bacterial adhesion, and has been associated with bacterial dissemination across intestinal and blood-brain barriers, leading to meningitis in neonates [16]. While our data does not permit to predict how the virulence of the ST297 strain would compare to that of ST1 or ST17 strains in a neonatal model of infection, interestingly, acquisition *hvgA* did not increase the virulence of the ST297 mosaic strain in a model of adult GBS disease. Indeed, this strain showed higher virulence in intraperitoneal-infection model than an *hvgA*-positive ST17 strain, but it was less virulent than an ST1 *hvgA*-negative strain. The mosaic strain also showed lower bacteremia than the ST1 strain at 6 h and 12 h p.i., indicating that *hvgA* does not contribute to bacterial dissemination when accompanying an ST1 genomic background and/or in the context of the adult mouse model used. The latter hypothesis seems more likely, as an *hvgA*-positive ST17 strain (NGBS128) had the lowest virulence profile. This suggests that HvgA is not essential for GBS virulence in adults. Other factors present in the recombined area (such as *cspA*, *cfb*, *rgfCA*, *dltABCD*) might be more relevant for virulence in an adult model of invasive GBS disease and/or during the septic shock phase of GBS disease. One limitation of our study is that we used only one serotype V ST297 strain. Assessment of the virulence of more recombinant isolates when they become available would be important to confirm this speculation. In addition, because strains tested here are non-isogenic, ascertaining the contribution to virulence of each of the ST17-derived factors is challenging, due to the significant background effects of the large area of recombination.

It is well recognized that the exacerbated inflammatory response induced early during the systemic phase of GBS infection is mostly responsible for the clinical outcome, including sudden death [26,27]. Yet, under normalized bacteremia levels, the ST297 and the highly virulent ST1 strains had similar intrinsic capacity to induce an inflammatory cascade of cytokines and chemokines. Thus, the diminished pathogenic capacity of the ST297 strain may be related to a reduced bacteremia when compared to ST1-infected mice. In contrast, the lower pathogenicity of the ST17 strain appears to be, at least in part, related to a reduced capacity to induce expression of key mediators of inflammation and septic shock, namely IL-1β, IL-6 and CXCL1. In a GBS-infection model, IL-1β deficiency has been associated with selective impairment in the production of the neutrophil chemokine CXCL1 [28]. This is an agreement with our data showing decreased IL-1β production concomitant to reduced CXCL1 release during infection with the ST17 strain. Increased systemic and local levels of IL-1 β and IL-6, together with tumor necrosis factor alpha, are known to correlate with a more severe development of sepsis in mice [29]. Furthermore, very high cytokine concentrations, such as IL-1β and IL-8, are found in septic newborn infants [26]. Thus, reduced levels of these inflammatory mediators might improve or delay the clinical outcome of ST17-infected animals compared to ST1- or ST297-infected ones. Interestingly, and in contrast to above-mentioned pro-inflammatory mediators, the CCL3 chemokine was highly upregulated in ST17-infected mice. In a number of model systems, CCL3 has been shown to play an important role in the recruitment of mononuclear cells [30,31]. In a *Klebsiella pneumoniae* infection model, CCL3 was shown to promote bacterial phagocytosis and killing, thus contributing to pathogen clearance [32]. The clinical course of disease, number of circulating bacteria, and systemic and local inflammation are all interconnected, and under complex regulation mechanisms that seem to differ between the different GBS STs [26,33].

Recombination in GBS has long been known to play important roles in the emergence of virulent strains causing disease in humans. Here, we show that the genome of strain NGBS375 (ST297) is a mosaic resulting from a recombination event involving acquisition of approximately 300 Kbp of ST17 genetic material in an ST1 genomic background, leading to reduced virulence in a murine model of adult disease. A better understanding of the dynamic expression of virulence traits in the different GBS genomic backgrounds, and of the cellular immunology in the context of GBS adult infection, is required to elucidate adult GBS disease pathogenesis.

## 4. Materials and Methods

### 4.1. Bacterial Strains and Growth Conditions

GBS strains NGBS375 (serotype V, ST297), NGBS357 (serotype V, ST1), and NGBS128 (serotype III, ST17) were used in this study. The ST297 and ST1 strains were isolated from the blood of unrelated adult patients in Toronto, Canada, in 2011 [15]. Strain NGBS128 (ST17) was isolated in 2010 in Toronto, Canada, from a case of late onset disease [2,15]. Strains were grown in Todd Hewitt broth (THB) or agar (THA) (Becton Dickinson, Mississauga, ON, Canada) or on sheep blood agar plates at 37 °C for 18 h. Inocula for in vivo infections were prepared as described elsewhere [34]. Briefly, GBS colonies were inoculated in THB and incubated at 37 °C with shaking for 8 h, then 10 μL of a 1/1000 dilution of the 8 h-cultures were inoculated into 30 mL of THB followed by incubation with shaking for 12 h at 37 °C. Bacteria were then washed and resuspended in 20 mL of THB to obtain an OD_600 nm_ of 0.5, which corresponds to approximately 2 × 10^8^ CFU/mL. Final suspensions were enumerated by plating appropriate dilutions onto THA.

### 4.2. Genome Sequencing and Analysis

The genomes of strains NGBS375 (ST297) and NGBS357 (ST1) were sequenced using PacBio sequencing (Pacific Biosciences, Menlo Park, CA, USA) as previously described [35]. Two cells of sequence were generated for each isolate. To assess base-calling accuracy in the PacBio assembly, Illumina short-reads for the two strains were generated. Genomic libraries were prepared using Nextera XT kits (Illumina, San Diego, CA, USA) and sequenced as paired-end reads in an Illumina HiSeq 2500 or MiSeq instrument (Illumina). Both genome assemblies were completely concordant with full length perfectly aligning Illumina short-reads. The genome of NGBS375 (ST297) was circularized. Circularization of NGBS357 could not be achieved due to the presence of a repetitive region of >80 kb in its genome. However, a single contig was obtained (Supplementary Figure S1). Genome assemblies are available in GenBank under Accession numbers CP012504 (NGBS357, ST1), and CP102503 (NGBS375, ST297). Supplementary Table S1 presents genome closure statistics. The genome sequences of strains NGBS128 (ST17), NGBS572 (ST452), NEM316 (ST23), A909 (ST7), NGBS061 (ST459), 2603VR (ST110) were retrieved from GenBank (Accession numbers CP012480, CP007632, AL732656, NC_007432, CP007631, and NC_004116, respectively). Short-read Illumina data for strain NGBS128 were retrieved from NCBI sequence-read archive (SRA, accession number SAMN04007140). Identification of tRNA-encoding regions and annotation were performed with Prokka [36]. Ortholog analysis was performed using PGAP [37]. Polymorphisms relative to strain NGBS375 were identified using VAAL [38] for strains for which Illumina data was available, otherwise with Mauve [39]. Recombination analysis was performed using BRATNextGen [18] run with 20 iterations and 100 replicates, with a *p*-value of 0.05 as the significance cut-off. Genome visualizations were created using BRIG [40]. Short reads have been made available at SRA under Study Accession PRJNA295774 and PRJNA274384 for the ST17 and ST1 strains, respectively.

### 4.3. Mouse Experimental Infections

All animal work was approved by the Animal Welfare Committee of the University of Montreal. Female, 5-6-week-old, C57BL/6 mice (Charles River Laboratories, Wilmington, MA, USA) were acclimatized to a 12-h-light/12-h-dark cycle with free access to food and water. On the day of the experiment, animals were divided into 4 groups. Group 1 (*n* = 15 mice) received 0.5 mL of an NGBS375 (ST297) suspension (10^5^ CFU/mL) by intraperitoneal injection. Groups 2 and 3 (*n* = 15 each) received the same dose of strains NGBS357 (ST1) and NGBS128 (ST17), respectively. Group 4 (*n* = 7) received 0.5 mL of sterile THB. Three independent preliminary trials were performed to establish the optimal bacterial doses and time points (data not shown). Mice were monitored daily to record clinical signs such as depression, rough appearance of hair coat, and swollen eyes, and/or mortality. Numbers of viable bacteria in blood were quantified at 6 h, 12 h and 24 h p.i. Briefly, blood samples (8 μL) were collected from the tail vein, serially diluted in PBS and plated onto THA plates as described above, and colonies enumerated. A second experiment was carried out to contrast the virulence of strains NGBS128 (ST1, *n* = 15 mice) and NGBS375 (ST297, *n* = 15 mice) at a higher infectious dose (10^7^ CFU). Experiments were carried out as described above. To measure plasma cytokine levels, mice were infected (intraperitoneal route) with 10^7^ CFU of the different GBS strains (*n* = 10 for each strain in two independent experiments). Mock-infected mice were used as negative controls. Mice were euthanized at 6 h p.i. and blood collected by cardiac puncture. Bacteremia levels were quantified as described above and plasma conserved at −80 °C for cytokine analyses.

### 4.4. Cytokine Quantification by ELISA

Levels of IL-1β, IL-6, CCL3 (MIP-1α), CXCL1 (KC), CXCL9 (MIG) and CXCL10 (IP-10) in the plasma of infected mice were measured by sandwich ELISA using pair-matched antibodies from R&D Systems (Minneapolis, MN, USA), according to the manufacturer’s recommendations. Twofold dilutions of recombinant mouse cytokines were used to generate standard curves. Sample dilutions giving OD readings in the linear portion of the appropriate standard curve were used to quantify the levels of each cytokine.

### 4.5. Statistical Analyses

Data from experimental infections are presented as Kaplan-Meier survival curves, mean ± SEM or geometric mean with 95% confidence interval where appropriate. Prism v.5 (Graphpad, San Diego, CA, USA) was used for data analysis. Log-rank (Mantel-Cox) tests were used to compare the survival curves. For bacteremia and plasma cytokine levels, an ANOVA test was performed. *p* < 0.05 was considered as the cut-off for statistical significance.

## Figures and Tables

**Table 1:** Number of single-nucleotide polymorphisms (SNPs) identified relative to ST297 strain NGBS375.

Strain	ST ^a^	No. of SNPs ^b^
NGBS572	452	15,244
NEM316	23	13,951
A909	7	13,800
NGBS061	459	11,142
2603V/R	110	10,987
NGBS128	17	21,022
NGBS357	1	2645

^a^ ST: Sequence type; ^b^ SNP: Single-nucleotide polymorphism.

## Supplementary Materials

The following files are available online at www.mdpi.com/2076-0817/5/3/54/s1, Figure S1: Genome atlas of the complete genome of NGBS357 (ST1), Table S1: Whole-genome sequencing statistics for genome closure, Table S2: Gene content in recombined region of NGBS375 (ST297), Table S3: Unique gene clusters found in NGBS375 (ST297), Table S4: Unique genes in NGBS128 (ST17), Table S5: Unique genes in NGBS357 (ST1).
