# Supplementary material for: Genomic Recombination Leading to Decreased Virulence of Group B Streptococcus in a Mouse Model of Adult Invasive Disease
Source: Pathogens. 2016 Aug 5;5(3):54. doi: 10.3390/pathogens5030054 (PMC5039434; doi:10.3390/pathogens5030054)
Supplement: Supplementary file 1 [file pathogens-05-00054-s001.pdf]

Supplementary Materials: Genomic Recombination Leading to Decreased Virulence of Group B *Streptococcus* in a Mouse Model of Adult Invasive Disease

Sarah Teatero, Paul Lemire, Ken Dewar, Jessica Wasserscheid, Cynthia Calzas, Gustavo V. Mallo, Aimin Li, Taryn B.T. Athey, Mariela Segura and Nahuel Fittipaldi

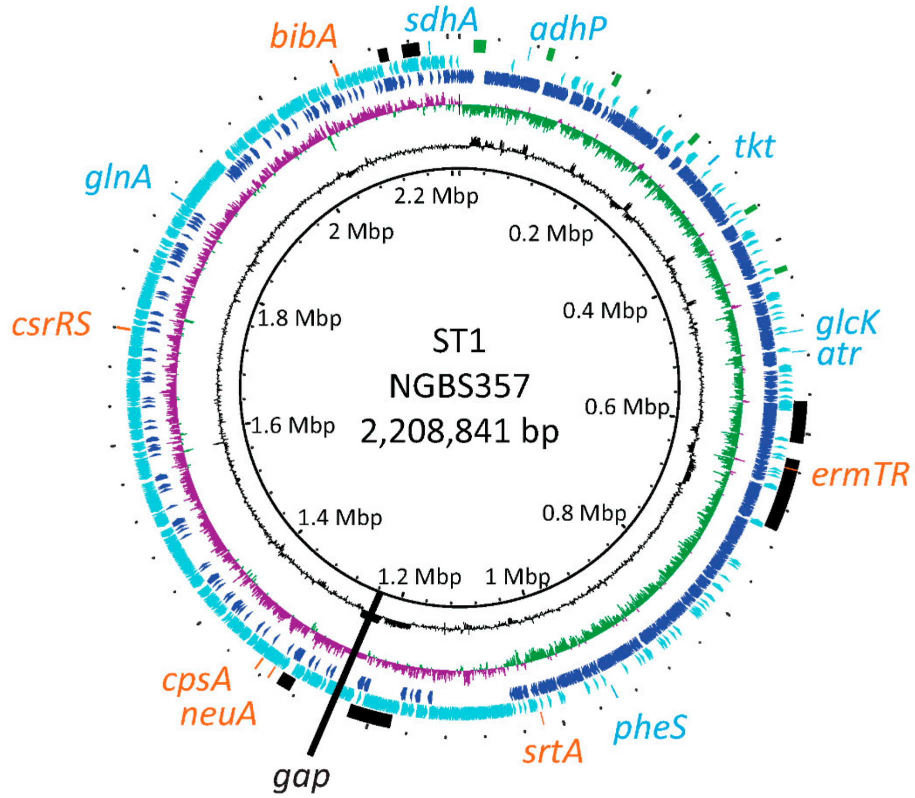

Figure S1. Genome atlas of the complete genome of NGBS357 (ST1). Depicted data from innermost to outermost circles represent genome size in Mbp (circle 1), percent G+C content (Circle 2), GC skew, where (G-C)/(G+C) is averaged over a moving window of 10,000 bp, with excess C shown in green and purple, respectively (circle 3). Circle 4 shows annotated coding sequences (CDSs) on the forward strand (**dark blue**), while circle 5 shows reverse-strand encoded CDSs (**light blue**). Reference landmarks (circle 6): mobile genetic elements, **black**; RNA, **green**; MLST genes, **light blue**; *bibA* (*hvgA* homologue) and other genes of interest, **orange**.

Table S1. Whole Genome Sequencing statistics for genome closure.

Strain	Accession Number	No. of Reads >3 kb (PacBio) (bp)	Ave. Length of Reads (PacBio) (bp)	Coverage of Final Assemblies (PacBio)	No. of Reads (Illumina)	Length of Reads (Illumina)	Coverage of Final Assemblies (Illumina)	Genome Size
NGBS375	CP012503	62,706	6087	176	2,250,144	150	155	2,172,875
NGBS357	CP012504	45,296	8119	166	2,146,206	101	98	2,208,841 [a]

[a] Size of sequenced contig. Does not include repetitive region.

Table S2. Gene content in recombined region of NGBS375 (ST297).

Strand	Position (Start End)	Gene	Product
-	1749923 1750483	*efp*	elongation factor P
-	1751411 1751710		hypothetical protein
-	1752128 1752358		hypothetical protein
-	1752371 1752541		hypothetical protein
-	1752554 1752994		hypothetical protein
-	1753598 1754050	*comEB*	late competence protein required for DNA binding and uptake
-	1754062 1755129		X-Pro aminopeptidase
-	1755229 1755945		membrane protein
-	1755947 1757446		EmrB/QacA family drug resistance transporter
-	1757782 1760610	*uvrA*	excinuclease ABC subunit A
-	1760742 1761413		hypothetical protein
-	1761438 1762382		magnesium transporter CorA family protein
-	1762552 1762791	*rpsR*	30S ribosomal protein S18
-	1762959 1764278		prophage LambdaSa1, reverse transcriptase/maturase family protein
-	1764859 1765350	*ssb-3*	single-stranded DNA-binding protein
-	1765362 1765649	*rpsF*	30S ribosomal protein S6
+	1766743 1767867	*mutY*	A/G-specific adenine DNA glycosylase
+	1768044 1768637		transcriptional regulator
-	1768683 1768997	*trx*	thioredoxin
-	1769078 1769578		phosphatidylglycerophosphatase B
-	1769582 1771921	*mutS2*	DNA mismatch repair protein mutS
-	1772006 1772548		CvpA family protein
-	1772551 1772862		Cell division protein ZapA
+	1772988 1773866	*rnhC*	ribonuclease HIII
+	1773882 1774475		signal peptidase I
+	1774604 1777024		helicase
+	1777138 1777620		membrane protein
-	1777691 1778800	*dinP*	DNA polymerase IV
+	1778970 1781282	*pf1*	pyruvate formate-lyase
+	1781383 1781580		FMN-binding protein
+	1781577 1781756		FMN-binding protein
-	1781791 1782720		beta-lactamase
-	1782717 1783472		C3-degrading proteinase
+	1783597 1784493		membrane protein
-	1784572 1785420		glycerol uptake facilitator protein
-	1785588 1786040		universal stress protein
-	1786058 1787260		transporter
+	1787416 1788075		Crp/Fnr family transcriptional regulator
+	1788099 1790384	*pepX*	x-prolyl-dipeptidyl aminopeptidase
+	1790388 1790747		hypothetical protein
+	1790793 1791773		polyprenyl synthetase
-	1791858 1793606	*cydC*	ABC transporter ATP-binding protein
-	1793599 1795317		ABC transporter ATP-binding protein
-	1795317 1796336	*cydB*	cytochrome d ubiquinol oxidase, subunit II
-	1796337 1797764	*cydA*	cytochrome d oxidase subunit I
-	1797867 1799075		pyridine nucleotide-disulfide oxidoreductase family protein
-	1799088 1799987		1,4-dihydroxy-2-naphthoate octaprenyltransferase
-	1800396 1800923		hypothetical protein
+	1801885 1802184		YbaB/EbfC family DNA-binding protein
+	1802344 1803534	*cfa*	cyclopropane-fatty-acyl-phospholipid synthase
-	1804069 1804329		MerR family transcriptional regulator
-	1804456 1805043		DNA polymerase III subunit epsilon
-	1805087 1805623		transcriptional regulator OrfX
+	1805758 1806930		putative flavoprotein

-	1806974 1807738		hypothetical protein
+	1807915 1808184	*rpsN_2*	30S ribosomal protein S14
-	1808545 1809570	*ltaE*	threonine aldolase
-	1809690 1810700		DNA-binding/iron metalloprotein/AP endonuclease
-	1810776 1811183		ribosomal-protein-alanine acetyltransferase
-	1811185 1811877		M22 peptidase
+	1812056 1812286		hypothetical protein
+	1812340 1814019		Zn-dependent hydrolase
-	1814177 1814686		CHAP domain-containing protein
-	1814834 1816180	*glnA*	glutamine synthetase, type I
-	1816214 1816585	*glnR*	GlnR family transcriptional regulator
-	1816665 1817204		signal peptide containing protein
-	1817467 1818663	*pgk*	phosphoglycerate kinase
-	1818798 1819667		acid phosphatase
-	1819876 1820886	*gapA*	glyceraldehyde-3-phosphate dehydrogenase
-	1821090 1823168	*fusA*	elongation factor G
-	1823323 1823793	*rpsG*	30S ribosomal protein S7
-	1823815 1824228	*rpsL*	30S ribosomal protein S12
-	1824447 1825259	*purR*	pur operon repressor
-	1825356 1826297		HD domain-containing protein
-	1826287 1827561		RmuC domain-containing protein
-	1827563 1828195		thiamine pyrophosphokinase
-	1828188 1828850	*rpe*	ribulose-phosphate 3-epimerase
-	1828857 1829729		ribosome-associated GTPase
-	1829885 1830736		rRNA (guanine-N1-)-methyltransferase
-	1830740 1831612	*ksgA*	dimethyladenosine transferase
-	1831692 1832054		hypothetical protein
-	1832202 1832762		primase-like protein
-	1832749 1833603		TatD family deoxyribonuclease
-	1833713 1833985		hypothetical protein
-	1834004 1834396		hypothetical protein
-	1834387 1835679		lipase
-	1835679 1836071		membrane protein
-	1836246 1837508	*dltD*	dltD protein
-	1837501 1837740	*dltC*	D-alanine--poly(phosphoribitol) ligase subunit 2
-	1837755 1839020	*dltB*	dltB protein
-	1839017 1840552	*dltA*	D-alanine--poly(phosphoribitol) ligase subunit 1
-	1840565 1840687	*dltX*	D-Ala-teichoic acid biosynthesis protein DltX
-	1840698 1841885		sensor histidine kinase
-	1841885 1842559		DNA-binding response regulator
-	1842915 1843049	*rpmH*	50S ribosomal protein L34
-	1843233 1844588		Nucleoside recognition
+	1844820 1845986		IS30 family transposase
-	1846281 1848008		amino acid ABC transporter permease
-	1848027 1849250		amino acid ABC transporter ATP-binding protein
-	1849627 1852005	*xfp*	phosphoketolase
-	1852095 1853186		L-ascorbate 6-phosphate lactonase
-	1853503 1855182		BglG family transcriptional antiterminator
-	1855248 1856009		Xylose isomerase-like TIM barrel
-	1856029 1857546		carbohydrate kinase
-	1857612 1858601		putative metal-dependent hydrolase of the TIM-barrel fold protein
-	1858623 1860074		PTS system transporter subunit IIC
-	1860172 1861128		glyoxylate reductase, NADH-dependent
-	1861147 1862166		hypothetical protein
-	1862401 1863384		LacI family sugar-binding transcriptional regulator
-	1863547 1864194		translaldolase
-	1864205 1864921	*araD*	L-ribulose-5-phosphate 4-epimerase

-	1864923 1865786		L-xylulose 5-phosphate 3-epimerase
-	1865790 1866455	*ulaD*	3-keto-L-gulonate-6-phosphate decarboxylase
-	1866568 1867053		PTS system transporter subunit IIA
-	1867120 1867398		PTS system 3-keto-L-gulonate specific transporter subunit IIB
-	1867426 1868865	*ulaA_2*	PTS system ascorbate-specific transporter subunit IIC
-	1869033 1869650		membrane protein
+	1869827 1870282		hypothetical protein
-	1870305 1871597	*purA*	adenylosuccinate synthetase
-	1871967 1872989	*pfoR*	perfringolysin O regulator protein
+	1873351 1874025		DNA-binding protein
+	1874195 1876447		bifunctional glutamate--cysteine
+	1876571 1877389		membrane protein
+	1877386 1878642		toxic anion resistance protein
+	1878782 1879657	*hslO*	heat shock protein 33
+	1879641 1880618		NifR3/Smm1 family protein
+	1880739 1881380		deoxynucleoside kinase family protein
+	1881394 1881885	*pat*	phosphinothricin *N*-acetyltransferase
-	1882105 1884552		ATP-dependent Clp protease, ATP-binding subunit
-	1884549 1885013	*ctsR*	CtsR family transcriptional regulator
+	1885186 1885647	*qacE*	putative small multi-drug export
-	1885806 1886846	*tsf*	elongation factor Ts
-	1886940 1887710	*rs2*	30S ribosomal protein S2
+	1887987 1888547	*ahpC*	alkyl hydroperoxide reductase
+	1888565 1890097	*ahpF*	alkyl hydroperoxide reductase
+	1890231 1890301		tRNA-Cys
-	1890374 1892443		Na+/H+ exchanger family protein
-	1892445 1892681		hypothetical protein
+	1892848 1893801		microcin immunity protein MccF
-	1893851 1895746	*pepO_2*	endopeptidase O
+	1895894 1896877		Gfo/Idh/MocA family oxidoreductase
-	1896923 1898086		putative inositol transporter 2
+	1898198 1898842		cyclic nucleotide-binding protein
+	1898908 1899522	*def*	peptide deformylase
-	1899594 1900595	*regR*	RegR family transcriptional regulator
-	1900675 1902579		oligohyaluronate lyase
-	1902659 1903474		PTS system transporter subunit IID
-	1903461 1904327		PTS system transporter subunit IIC
-	1904363 1904857		PTS system transporter subunit IIB
-	1904912 1906108		glucuronyl hydrolase
-	1906111 1906545		PTS system transporter subunit IIA
+	1906823 1907635		gluconate 5-dehydrogenase
+	1907652 1908290		ribose/galactose isomerase
+	1908316 1909323		carbohydrate kinase
+	1909335 1909973	*eda-2*	keto-hydroxyglutarate-aldolase/keto-deoxy-phosphogluconate aldolase
+	1911036 1912532		ribonucleases G and E
-	1912704 1913318		nitroreductase family protein
-	1913451 1913876		MarR family transcriptional regulator
-	1914003 1918409	*polC*	DNA polymerase III PolC
-	1918532 1919116		*N*-acetylmuramoyl-L-alanine amidase
-	1919241 1921094	*proS*	prolyl-tRNA synthetase
-	1921186 1922445		zinc metalloprotease
-	1922476 1923270	*cdsA*	phosphatidate cytidylyltransferase
-	1923285 1924037	*uppS*	undecaprenyl pyrophosphate synthase
-	1924204 1924545	*yajC*	preprotein translocase subunit YajC
-	1924622 1924966		bacterocin transport accessory protein
-	1925157 1926320		malate oxidoreductase
-	1926345 1927682		CCS family citrate carrier protein

+	1927836 1929362		sensor histidine kinase
+	1929364 1930044		response regulator
-	1930065 1931060	*galE*	UDP-glucose 4-epimerase
-	1931148 1932755	*dexB*	glucan 1,6-alpha-glucosidase
-	1932884 1934017		sugar ABC transporter ATP-binding protein
-	1934118 1934969		helix-turn-helix domain-containing protein
-	1935064 1935960		aldolase 1 epimerase LacX
-	1936014 1936991	*lacD*	tagatose 1,6-diphosphate aldolase
-	1936993 1937802	*lacC_1*	tagatose-6-phosphate kinase
-	1937884 1938717		IS861, transposase OrfB
-	1938693 1939229		IS861, transposase OrfA
-	1939262 1939360	*lacC_2*	tagatose-6-phosphate kinase
-	1939371 1939886	*lacB*	galactose-6-phosphate isomerase subunit LacB
-	1939984 1940331	*lacA*	galactose-6-phosphate isomerase subunit LacA
-	1940580 1943030		neuraminidase-like protein
-	1943381 1943758		PTS system transporter subunit IIC
-	1943798 1944103		PTS system transporter subunit IIB
-	1944105 1944569		PTS system transporter subunit IIA
+	1944787 1945563	*lacR-2*	lactose phosphotransferase system repressor
-	1945844 1948426		streptococcal histidine triad family protein
-	1948453 1949376		adhesion lipoprotein
-	1949631 1950074		D-tyrosyl-tRNA(Tyr) deacylase
-	1950084 1952300		GTP pyrophosphokinase
+	1952560 1954962	*cpdB*	bifunctional 2′,3′-cyclic nucleotide 2′-phosphodiesterase/3′-nucleotiase precursor protein
+	1955119 1955574	*nrdI-2*	flavoprotein NrdI
+	1955585 1956622		M42 family peptidase
+	1956622 1957119		polynucleotide kinase
+	1957176 1957295		hypothetical protein
-	1957341 1958378		iron ABC transporter substrate-binding protein
-	1958390 1959163		DNA-binding response regulator
-	1959167 1960816	*yesM*	two-component sensor kinase YesM
-	1960954 1961781		phospotransferase system (PTS), enzyme II component D
-	1961778 1962587		PTS system transporter subunit IIC
-	1962591 1963094		PTS system transporter subunit IIB
-	1963112 1963537		PTS system transporter subunit IIA
-	1963784 1964818	*alr_2*	alanine racemase
-	1965047 1966201		membrane protein
-	1966207 1967055		ABC transporter ATP-binding protein
+	1967586 1967768		ABC transporter ATP-binding protein
-	1967839 1969173	*rgfC*	histidine kinase
-	1969170 1969922		response regulator
-	1970138 1970953		endonuclease/exonuclease/phosphatase family protein
-	1971007 1973190		PTS system transporter subunit IIABC
-	1973345 1975000		sensor histidine kinase
-	1974993 1975670	*phoB*	phosphate regulon response regulator PhoB
-	1975670 1976326	*phoU*	phosphate transport system regulatory protein PhoU
-	1976323 1977072		phosphate transporter ATP-binding protein
-	1977065 1977943		phosphate ABC transporter permease
-	1977945 1978790		phosphate ABC transporter permease
-	1978805 1979686		hemolysin
-	1979887 1980171		hypothetical protein
-	1980168 1980473		hypothetical protein
-	1980470 1981210		16S ribosomal RNA methyltransferase RsmE
-	1981210 1982163	*prmA*	50S ribosomal protein L11 methyltransferase
-	1982160 1982468		MepB protein
+	1982742 1983458		MerR family transcriptional regulator
-	1983497 1983967		acetyltransferase

-	1983939 1984397		MutT/nudix family protein
-	1984533 1985003		hypothetical protein
+	1985345 1985821		acetyltransferase
+	1985814 1987082		recombination factor protein RarA
+	1987364 1987436		tRNA-Lys
-	1987625 1987930		hypothetical protein
-	1987914 1988315		hypothetical protein
-	1988303 1989520		lipase
-	1989557 1989943		membrane protein
-	1990114 1990623		hypothetical protein
-	1990625 1990957		membrane protein
-	1991217 1991537		hypothetical protein
-	1991776 1992630		streptomycin resistance protein
-	1992809 1993201		hypothetical protein
-	1993643 1994251		membrane protein
-	1994238 1994573		PadR family transcriptional regulator
-	1995095 1995583		acetyltransferase
-	1995752 1996513	*yxlG*	putative transmembrane protein YxlG
-	1996510 1997412		ABC transporter ATP-binding protein
-	1997409 1997615		hypothetical protein
-	1997617 1998693		Cro/CI family transcriptional regulator
-	1998938 1999771		IS861, transposase OrfB
-	1999747 2000283		IS861, transposase OrfA
-	2000377 2000991		PAP2 family protein
+	2001205 2001522		MagZ family protein
+	2001519 2002079		decarboxylase
+	2002384 2003247		protease
-	2003404 2003706		rhodanese-like domain-containing protein
-	2003879 2004646	*cfb*	cAMP factor
+	2005416 2005955		topology modulation protein
+	2006156 2007241		glycerol dehydrogenase
-	2007406 2008113		cyclic nucleotide-binding protein
-	2008460 2010304		bifunctional homocysteine S-methyltransferase/5,10-methylenetetrahydrofolate reductase
-	2010349 2012586	*metE*	5-methyltetrahydropteroyltriglutamate—homocysteine S-methyltransferase
-	2012956 2013279	*azlD*	branched-chain amino acid transport
-	2013269 2013961	*azlC*	branched-chain amino acid transport protein AzlC
+	2014284 2018999	*cspA*	cell surface serine endopeptidase CspA
+	2019232 2019633		DNA-binding response regulator
+	2019684 2019917		DNA-binding response regulator
+	2020046 2021302		sensor histidine kinase
+	2021395 2022003		hypothetical protein
-	2022093 2024594	*leuS*	leucyl-tRNA synthetase
+	2024810 2025976		IS30 family transposase
+	2026418 2027665		major facilitator family protein
-	2027708 2028553		alpha-beta hydrolase superfamily esterase
+	2028758 2029954		glycosyl transferase family protein
+	2030019 2031224		glycosyl transferase family protein
-	2031271 2031810	*nusG*	transcription antitermination protein NusG
-	2032016 2033800	*hvgA*	peptidoglycan linked protein
+	2033928 2034311		transposase protein A
+	2034344 2034733		transposase protein B
-	2034975 2035148	*secE*	preprotein translocase subunit SecE
-	2035184 2035336	*rpmG_1*	50S ribosomal protein L33
-	2035385 2037706	*pbp2A*	penicillin-binding protein 2A
+	2037750 2038634		ribosomal large subunit pseudouridine synthase D
-	2038659 2040299		SPBc2 prophage-derived transglycosylase YomI

-	2040469 2041680	*deoB_2*	phosphopentomutase
-	2041747 2042418	*deoC*	deoxyribose-phosphate aldolase
-	2042448 2043650	*nupC*	nucleoside permease nupC
-	2043671 2044450	*udp*	uridine phosphorylase
+	2044610 2045347		GntR family transcriptional regulator
+	2045360 2045656		hypothetical protein
-	2045756 2047378	*groEL*	Chaperonin GroEL (HSP60 family)
-	2047474 2047758	*groES*	co-chaperonin GroES
-	2047933 2048736		ABC transporter ATP-binding protein
-	2048741 2049637		ABC transporter permease
-	2049653 2050615		ABC transporter substrate-binding protein
-	2051080 2051877		HAD superfamily hydrolase
+	2052063 2052923		glyoxylase
+	2052966 2053697		YaaA-like protein
-	2054054 2054671	*nrdG*	anaerobic ribonucleoside-triphosphate reductase activating protein
-	2054744 2055235		acetyltransferase
-	2055244 2056176		virulence factor MviM
-	2056189 2056332		hypothetical protein
-	2056407 2058605		anaerobic ribonucleoside triphosphate reductase
-	2058704 2060245	*ccs4*	Competence-induced protein Ccs4

Table S3. Unique gene clusters found in NGBS375 (ST297).

Strand	Start	End	Gene	Product
-	516248	516799		phage protein
-	516817	517203		phage protein
-	517207	517554		Cro/CI family phage transcriptional regulator
+	517850	518095		phage protein
-	518046	518834		phage membrane protein
+	518885	519076		phage DNA-binding protein
+	519156	519467		phage protein
+	519615	519842		phage protein
+	519835	520065		hypothetical protein
+	520049	521368		chromosome segregation ATPases
+	521383	522456		phage protein
+	522552	523157		phage protein
+	523157	523765		hypothetical protein
+	523762	525354		DNA/RNA helicase
+	525363	525560		hypothetical protein
-	525553	525804		hypothetical protein
+	525875	528154		ATPase
+	528524	528721		phage protein
+	528748	529146		phage protein
+	529143	529358		phage associated protein
+	529355	529591		phage protein
+	529821	530153		hypothetical protein
+	530231	530644		phage protein
+	530765	531196		Phage terminase small subunit
+	531186	532466		terminase large subunit
+	532481	534010		phage portal protein
+	533976	535418		phage protein
+	535518	535634		phage membrane protein
+	535639	535842		phage protein
+	535985	536554		phage protein
+	536573	537469		phage protein
+	537475	537831		phage protein
+	537842	538120		phage protein
+	538117	538461		phage protein
+	538465	538824		phage protein
+	538836	539468		phage major tail protein
+	539519	539974		phage protein
+	540049	540279		phage protein
+	540308	544534		phage minor tail protein
+	544547	545389		phage protein
+	545402	549229		phage protein
+	549238	549390		hypothetical protein
+	549401	549817		phage protein
+	549817	550041		hypothetical protein
+	550051	550347		phage membrane protein
+	551747	552706		abortive infection bacteriophage resistance protein
+	619252	619533		membrane protein
+	620391	621242		DNA replication protein
+	621239	621724		hypothetical protein
+	621721	623505		type IV secretory pathway protein VirD4

+	623559	623690		hypothetical protein
+	623671	623982		single-strand binding protein
+	623986	624117		conjugative transposon membrane protein
+	624845	626722		prophage LambdaSa1, reverse transcriptase/maturase family protein
+	626992	627855		membrane protein
+	627870	628136		hypothetical protein
+	628441	630870		conjugal transfer protein
+	630875	633007		membrane protein
+	633020	633256		hypothetical protein
+	633234	635420		membrane protein
+	635518	637224		DNA topoisomerase
+	637306	638841		transcriptional regulator
+	638924	647680		helicase
+	647723	648382		conjugative transposon protein
+	648811	649584		ABC transporter ATP-binding protein
+	649574	651628		putative ABC transporter
+	651661	652344		DNA-binding response regulator
+	652334	653332		signal transduction histidine kinase
-	653364	654695		relaxase/mobilisation protein
-	654698	655054		conjugative transposon mobilization protein
+	655468	656745		transposase
+	657017	657988		AraC family transcriptional regulator
+	658151	658753	*eqbH*	ABC transporter permease
+	658757	659443		cobalt ABC transporter permease
+	659458	660924	*eqbJ*	ABC transporter ATP-binding protein
+	660940	662649	*eqbK*	ABC transporter ATP-binding protein
+	662650	664395		ABC transporter ATP-binding protein
+	664421	665773		Na+ driven multidrug efflux pump
+	666087	666497		DNA-binding protein
+	666958	667104		hypothetical protein
+	667166	668953		resolvase family site-specific recombinase
-	670192	671409		Tn916, transposase
-	671491	671694		Tn916, excisionase
-	683937	684158		Tn916 hypothetical protein
+	778487	779278		proteinase
+	1091341	1092285		ABC transporter substrate-binding protein
+	1092282	1093004		ABC transporter substrate-binding protein
-	1172486	1172656		fibrinogen-binding protein
-	2035184	2035336	*rpmG_1*	50S ribosomal protein L33
-	2080949	2081470		cytoplasmic protein
-	2081568	2083052		phage integrase family site specific recombinase
-	2083062	2083310		excisionase
-	2083376	2084404		replication initiation factor
-	2084407	2084838		hypothetical protein
-	2085114	2085569		hypothetical protein
-	2085569	2085784		hypothetical protein
+	2086107	2087027		Cro/CI family transcriptional regulator
+	2087048	2088169		phage protein, Fic family
+	2088274	2089083		hypothetical protein

Table S4. Unique genes in NGBS128 (ST17).

Strand	Start	End	Gene	Product
+	66832	68076		phage protein
+	68208	69053		hypothetical protein
+	80534	81196	*thrC_2*	threonine synthase
+	104048	104479		lipoprotein
+	104903	105343		lipoprotein
+	105336	105680		hypothetical protein
-	130075	131241		IS30 family transposase
+	155924	156325		membrane protein
-	181915	182418		deoxyribonuclease
-	182757	182945		hypothetical protein
+	195707	196078	*cglA*	competence protein CglA
+	197612	197779		hypothetical protein
+	198046	198213		late competence protein ComGG
+	237415	237537		transcriptional regulator
-	239957	240475		immunity repressor protein
+	240913	241752		gp49 bacteriophage-like protein
+	241805	242104		bacterial mobilization protein
+	242112	242444		mobilisation protein
+	242425	242619		hypothetical protein
+	242931	243620		methylase
+	243625	247995		type II restriction endonuclease
+	248155	251910	*yobl*	DNA-binding protein
+	251936	253378		putative transcriptional regulator
+	254241	254462		MutR family transcriptional regulator
+	270599	270829		acetyltransferase
-	297413	297901		PTS system transporter subunit IIBC
+	354142	354414		competence protein F
+	435763	436080		protease
-	462328	462576		acetate kinase
-	462610	462825		methyltransferase
+	464415	464984		acetyltransferase
-	469098	469877		hypothetical protein
+	470877	472316		ATP-dependent RNA helicase
-	488413	488649		BioY family protein
-	539128	539505		putative phosphoesterase
-	539683	539988		putative phosphoesterase
+	615022	615345		ABC transporter ATP-binding protein
+	633557	634000		hypothetical protein
+	640188	640706		hydrolase
+	640663	641187		hydrolase
+	655947	656591		sugar transporter
+	752788	753069		GBSi1, group II intron, maturase
+	795967	796656		cell wall surface anchor family protein
+	883664	883984	*glgD_1*	glucose-1-phosphate adenylyltransferase, GlgD subunit
+	920583	920864		GBSi1, group II intron, maturase
+	937667	937927		hypothetical protein
+	982348	983121		hypothetical protein
+	985909	986181		hypothetical protein
+	986460	987221		hypothetical protein
+	987855	988880		hypothetical protein
+	995376	995651		Tn916 ORF13 protein
+	1000548	1001741		site-specific recombinase, phage integrase family
-	1007872	1008051		PnkB-like serine/threonine kinase protein
-	1045991	1046173		iron-compound ABC transporter iron-compound-binding protein

-	1058271	1058648		lipoprotein
-	1059243	1059623		lipoprotein
-	1059665	1059931		lipoprotein
-	1059928	1060335		hypothetical protein
-	1060298	1060531		hypothetical protein
-	1060610	1061086		hypothetical protein
-	1085752	1086408		fibrinogen-binding protein
-	1122915	1123085		short chain dehydrogenase/reductase oxidoreductase
+	1150593	1151021		polysaccharide deacetylase
-	1152506	1153051		transporter BCCT family protein
-	1192339	1193307	*cpsl_1*	capsular polysaccharide biosynthesis protein
-	1193304	1194449	*cpsL_2*	capsular polysaccharide polymerase
-	1243644	1243907		C4-dicarboxylate transporter/malic acid transport protein
-	1268236	1268517		GBSi1, group II intron, maturase
-	1275642	1276190		hypothetical protein
-	1276287	1276517		hypothetical protein
+	1276936	1277508	*tnpA*	transposase
+	1285361	1286521		hypothetical protein
-	1287829	1288638		type II restriction enzyme (Eco47II, Sau96I)
-	1288639	1289640	*dcm*	DNA (cytosine-5-)-methyltransferase
-	1326896	1327093		acetyltransferase
-	1366044	1366211		hypothetical protein
-	1369052	1369816	*secA*	preprotein translocase subunit SecA
-	1371446	1371991	*asp3*	accessory secretory protein
-	1408485	1408952		sortase
-	1409076	1409747		hypothetical protein
-	1409737	1410612		sortase
-	1410650	1412158		surface protein Spb1
-	1412199	1416503		Cna B domain-containing protein
-	1416523	1417092	*lepB*	signal peptidase I
-	1417076	1417348		DNA repair ATPase
-	1436872	1437705		IS861, transposase OrfB
+	1443788	1443937		hypothetical protein
-	1458568	1459629		hypothetical protein
-	1459735	1463892		subtilisin-like serine protease
-	1466187	1467107		protease
+	1476875	1478041		IS30 family transposase
-	1509738	1510058		peptide ABC transporter ATP-binding protein
+	1522307	1523140		IS861, transposase OrfB
-	1535827	1535928		glyoxylase
-	1557125	1557514		D-isomer specific 2-hydroxyacid dehydrogenase family protein
-	1626947	1627651		hypothetical protein
-	1628312	1629265		ketopantoate reductase PanE/ApbA superfamily protein
-	1629396	1629596		hypothetical protein
-	1629606	1629959		DNA/RNA non-specific endonuclease
-	1630026	1630301		hypothetical protein
-	1630337	1630450		hypothetical protein
-	1635862	1636035		MerR family transcriptional regulator
-	1760007	1760450		lipase
-	1892744	1892917		bacteriocin
+	1946432	1946923		sensor histidine kinase
+	1950431	1951597		IS30 family transposase
-	2044819	2044989		LysM domain-containing protein

Table S5. Genes unique to NGBS357 (ST1).

Start	Stop	Gene	Product
62226	62459		hypothetical protein
240407	241627		integrase
241688	241948		DNA-binding protein
241960	242706		Rep protein
243664	244197		hypothetical protein
244197	244535		hypothetical protein
244718	245647		XRE family transcriptional regulator
245644	245910		hypothetical protein
246056	247957	*sthlM*	type III restriction-modification system methylation subunit
247950	250649	*sthlR*	DNA endonuclease, type III restriction and modification system
329195	329602		histidine kinase
569802	570005		phage protein
570108	570665		Region found in RelA/SpoT proteins
570667	571398		Repressor protein
571771	571914		hypothetical protein
571911	572135		hypothetical protein
572194	572352		phage protein
572393	572530		hypothetical protein
572499	573194		hypothetical protein
573249	573458		phage protein
573447	573833		phage protein
573907	574125		DNA-binding phage protein
574136	574930		antirepressor
574963	575223		phage protein
575158	575964		phage protein
576014	576157		hypothetical protein
576105	576440		hypothetical protein
576585	576770		DNA-binding phage protein
576843	577073		hypothetical protein
577977	578759		phage DNA replication protein
578886	579161		hypothetical protein
579148	579402		phage protein
579567	580520		Recombinational DNA repair protein RecT (prophage associated)
580517	581314		phage protein
581483	581824		phage protein
581821	582333		phage protein
582320	582517		phage protein
582511	582795		phage protein
582792	583061		phage protein
583075	583491		phage protein
583776	583919		hypothetical protein
583916	584428		phage protein
584449	584748		phage membrane protein
584766	584939		phage protein
584936	585130		hypothetical protein
585127	585393		phage protein
585788	586213		phage protein
587036	587392		phage protein
587389	588657		structural phage protein
588650	589870		phage protein
589870	590058		hypothetical protein
590167	591582		phage terminase
591663	592127		phage protein
592130	593032		prophage LambdaSa1, structural protein
593029	593244		phage protein

593258	593680		phage protein
593640	593978		phage protein
593971	594207		phage protein
594208	594543		phage protein
594553	595110		prophage LambdaSa1, structural protein
595110	595355		phage protein
595370	595741		phage protein
595741	597753		prophage LambdaSa1, pblA protein, internal deletion
597747	599279		prophage LambdaSa03, tail component
599280	603095		prophage LambdaSa1, *N*-acetylmuramoyl-L-alanine amidase family protein
603106	605118		prophage LambdaSa1, minor structural protein
605132	605458		hypothetical protein
605433	605645		hypothetical protein
605658	605960		hypothetical protein
608206	608715	*int3_2*	DNA integration/recombination/inversion protein
636412	636891		BFD-like [2Fe-2S] binding domain protein
637479	638210	*erm(TR)*	dimethyl adenosine transferase
638635	639324		hypothetical protein
639340	639723		hypothetical protein
639927	640370		hypothetical protein
640373	640702		hypothetical protein
641175	642155		tyrosine recombinase XerC
642751	643905		hypothetical protein
644128	644484		hypothetical protein
644553	645149		hypothetical protein
645290	645463		hypothetical protein
645551	647224		Phage integrase (Site-specific recombinase)
984217	984855	*inlA_2*	putative internalin
1104962	1106065		DNA translocase FtsK
1230142	1231899		DNA polymerase III gamma and tau subunits C terminal
1231905	1232588		hypothetical protein
1232585	1232911		hypothetical protein
1232889	1235885		DNA polymerase III gamma and tau subunits C terminal
1235931	1236227		hypothetical protein
1239355	1239531		hypothetical protein
1239528	1240226		hypothetical protein
1240352	1240594		hypothetical protein
1385509	1385724		hypothetical protein
1766681	1767058		HAD superfamily hydrolase
1817239	1817613		FMN-binding protein
1837276	1838856	*hsdM*	type I restriction-modification system, M subunit
1839480	1841252		hypothetical protein
1841390	1843375	*tkt_2*	transketolase
1843424	1844068		translaldolase
1844086	1844775		allulose-6-phosphate 3-epimerase
1844787	1845890		PTS system fructose-specific transporter subunit IIC
1845903	1846352		PTS system fructose-specific transporter subunit IIA
1846366	1846677		PTS system fructose-specific transporter subunit IIB
1846691	1848580		PRD domain/PTS system IIA domain-containing protein
1848791	1849018		hypothetical protein
1849051	1849350		hypothetical protein
1849361	1849534		chromosome replication initiation inhibitor protein
1849654	1849755		short chain dehydrogenase
1850122	1850472		short chain dehydrogenase
1850478	1851215		2,5-diketo-D-gluconate reductase A
1851748	1852278		glutamate-rich protein GrpB
1852320	1853111		PhzF family phenazine biosynthesis protein
1853121	1853531		acetyltransferase

1856181	1856906		MerR family transcriptional regulator
1884220	1884669		HIT family protein
1991044	1992492		PTS system transporter subunit IIC
2033128	2033436		RNA-binding protein
2034023	2034550		membrane protein
2034553	2034741		transcriptional regulator
2039292	2039507		hypothetical protein
2040900	2041217		hypothetical protein
2041501	2041605		hypothetical protein
2041963	2042172	*esxA*	virulence factor EsxA
2042653	2042757		hypothetical protein
2042935	2043663		hypothetical protein
2043688	2043807		hypothetical protein
2043909	2044151		DNA-damage-inducible protein J
2044131	2044250		hypothetical protein
